# HHV-6 Meningoencephalitis in a Healthy Adult Female

**DOI:** 10.1155/2023/9622004

**Published:** 2023-04-18

**Authors:** Nicholas Valentini, Cynthia Chen

**Affiliations:** Department of Emergency Medicine, University of Michigan, USA

## Abstract

**Introduction:**

We describe the case of a 47-year-old female who presented to an academic tertiary emergency department with two to three days of worsening fever, headache, malaise, and rigors. A broad infectious workup revealed a diagnosis of Human Herpesvirus 6 (HHV-6) meningoencephalitis without any additional identifiable causes. HHV-6, the virus responsible for the childhood disease roseola, is a common cause of fever, seizures, diarrhea, and a characteristic faint-pink rash in children. Symptomatic HHV-6 infection in adults is far less common. We believe this represents one of only a few reported cases of HHV-6 meningoencephalitis in an immunocompetent host. *Case Report*. A 47-year-old female presented to the emergency department with two to three days of fever, headache, malaise, and rigors. She had a noncontributory medical, surgical, and family history but had traveled extensively in northeast Africa six months prior. A physical exam was notable for a wide based gait, photophobia, mild nuchal rigidity, and pain with active range of motion of the neck. A broad infectious workup was pursued; however, given headache, fever, and subjective nuchal rigidity, the highest concern was for meningoencephalitis. A lumbar puncture was positive for HHV-6 without any other diagnostic findings to otherwise explain the patient's symptoms. The patient was discharged on hospital day 3 with improving symptoms.

**Conclusion:**

HHV-6 meningoencephalitis has previously been described as a pathogen associated with individuals with immunosuppressive conditions. There have been several prior case reports of symptomatic meningoencephalitis in immune-competent individuals, and we believe this case adds to a growing body of evidence that HHV-6 meningoencephalitis can cause symptomatic infection in a broader patient population.

## 1. Introduction

We describe the case of a 47-year-old female who presented to an academic tertiary emergency department with two to three days of worsening fever, headache, malaise, and rigors. The patient had no relevant medical history or immunocompromising conditions but had traveled extensively within northeast Africa six months prior to her ED presentation. A broad infectious workup revealed a diagnosis of Human Herpesvirus 6 (HHV-6) meningoencephalitis without any additional identifiable causes. HHV-6 is more commonly known as the virus responsible for roseola, a childhood infection causing fever, rash, and diarrhea, with the rash commonly appearing later in the course of the illness. We believe this represents one of only a few reported cases of HHV-6 meningoencephalitis in an immunocompetent host. It is possible that HHV-6 meningoencephalitis may be an emerging pathogen of importance in causes of symptomatic central nervous system infections.

## 2. Case Report

A 47-year-old female presented to the emergency department with two to three days of fever, headache, malaise, and rigors. She had a stated past medical history of hypertension on metoprolol. She denied any other medical or surgical history. The patient did not endorse any additional medication or supplement use. She had no recent hospitalizations. She had traveled to several locations in northeast Africa six months prior to her emergency department presentation but did not have any symptoms during or immediately after her trip. She stated she was up to date on routine vaccinations; however, these records were not available at the time of this encounter.

A physical exam was notable for a wide based gait, photophobia, mild nuchal rigidity, and pain with active range of motion of the neck. Her Glasgow Coma Scale was 14, as she required verbal prompting to maintain alertness and to respond to questioning, but was otherwise oriented to person, place, time, and events leading to her presentation. She did not have any cardiopulmonary abnormalities. There were no rashes or other skin lesions, no abdominal tenderness, and no vomiting or diarrhea; however, she did endorse nausea. There was no report of urinary frequency or dysuria. She denied recent sexual activity. She denied environmental exposures in her home state, and she denied routinely performing work or recreational activities outdoors.

A broad infectious workup was pursued; however, given the headache, fever, and subjective nuchal rigidity, our highest concern was for meningoencephalitis. A lumbar puncture was performed, and in addition to standard cell count and culture, an arbovirus panel and viral PCR (polymerase chain reaction) were performed. Cerebrospinal fluid analysis was notable for clear fluid with 6 red blood cells and 1 white blood cell in tube 1, 0 red blood cells, and 1 white blood cell in tube 4, with a glucose of 53 and protein of 25. HSV, VZV, and cryptococcal testing was negative. The CSF viral PCR returned positive for HHV-6 without any other pertinent blood, urine, stool, or cerebrospinal fluid studies to otherwise explain the patient's symptoms. Notably, malaria (via blood smear testing), viral upper respiratory infection (via nasopharyngeal PCR testing), urinary tract infection (via urinalysis), and COVID-19 were also considered in our differential and were excluded by laboratory evaluation. A preliminary diagnosis of HHV-6 meningoencephalitis was made, and the patient was admitted for infectious disease consultation and supportive care.

The patient was discharged on hospital day 3 with improving symptoms, no longer febrile. A broadened workup in the hospital for immunocompromising conditions and travel-specific pathogens yielded no additional pertinent positive results.

## 3. Discussion

Human Herpesvirus 6 (HHV-6) is a member of the Betaherpesvirinae subfamily of herpes viruses. It is commonly detected in the pediatric population where it causes Roseola infantum (sixth disease) most often in children under three years of age [1]. This self-limited disease usually is treated with supportive care and resolves within 3-5 days. Many individuals have prior exposure to HHV-6 with some studies reporting seroprevalence approaching 100% [1]. Reactivation of HHV-6 may occur in immunocompromised patients such as those on immunosuppressive medication, long-term steroid therapy, chemotherapy, or those with human immunodeficiency virus (HIV) [2].

HHV-6 meningoencephalitis is a rare cause of central nervous system (CNS) symptoms in the immunocompetent population [2, 3]. While our patient had PCR-proven detection of HHV-6 in CNS fluid, there is some consideration regarding the diagnostic validity of this test. PCR detection of viral DNA was 92% sensitive in the identification of primary infection [3]. Laboratory guidelines for the definitive diagnosis of HHV-6 infection with CNS involvement call for both positive viral identification in the cerebrospinal fluid and blood and IgM detection [4]. Immunoglobulin testing was not performed during this patient encounter. Rarely, prior HHV-6 infection can be incorporated into a host's chromosomal DNA and cause persistent false positive PCR testing [4]. PCR detection alone is therefore unable to differentiate between active infection and persistent viral presence via chromosomal integration. However, this test is not routinely performed outside of specialized laboratory settings. Given the patient had symptomatology that fit with a primary CNS infection, we have high suspicion this does represent a case of HHV-6 meningoencephalitis.

Given the relatively low prevalence of HHV-6 meningoencephalitis, a definitive treatment strategy has not been established [5]. Multiple prior case reports emphasize the use of standard supportive care measures and inpatient hospitalization for monitoring. Antiviral therapies including foscarnet, cidofovir, and ganciclovir have all been trialed with ganciclovir showing the most benefit in terms of time to symptom relief [6]. However, there are multiple notes in the literature regarding significant side effects when foscarnet or cidofovir is utilized, and due to its overall favorable safety and side effect profile, ganciclovir appears to be the most recommended treatment strategy if antiviral therapy is being considered [6]. In the case of our patient, infectious disease was consulted later in the hospital course and ultimately no antiviral treatment was administered. The patient remained stable, without declining mental status, and without need for significant supportive care beyond intravenous fluids and oral antipyretic medications. Therefore, it was felt the possible risks of treatment, and the possible side effect profile of any of the considered medications would outweigh the potential benefit.

## 4. Conclusions

HHV-6 meningoencephalitis has previously been associated with individuals with immunosuppressive conditions. There have been several prior case reports of symptomatic CNS infections in immune-competent individuals, and we believe this case adds to a growing body of evidence that HHV-6 meningoencephalitis can cause symptomatic infection in a broader patient population. While our patient recovered with several days of supportive care, her travel history and initial clinical presentation prompted a broad infectious workup including consideration of pathogens typically not seen at our institution. As always, clinicians should maintain a high index of suspicion for atypical pathogens, particularly in medically complex patients or those with additional risk factors including environmental exposures or travel out of area. Attention to a good history of present illness as well as a thorough overall medical, social, and travel history is critical to the diagnostic evaluation. Specific to our case, HHV-6 meningoencephalitis should be considered alongside other possible CNS pathogens as a cause of illness even in immunocompetent patients.
